# 

**Published:** 1917-07-21

**Authors:** 


### £100,000 Red Cross Gift.

As a result of an appeal organised throughout the State of Victoria by Lady Stanley, wife of the Hon. Sir Arthur Stanley, the Governor and President of the Victoria Division of the Australian Red Cross, the magnificent sum of £100,000 has been handed to the Joint War Committee of the British Red Cross Society and the Order of St. John.

### Gas for Heating and Cooking in Hospitals.

The June number of *A Thousand and One Uses for Gas* is especially interesting to those engaged in hospital management, and contains articles on the use of gas as a factor of economy in the management of a hospital, by a hospital secretary, and, from the architect's point of view, by Mr. Thomas A. Pole, A.R.I.B.A. A number of excellent photographs and diagrams illustrate the cooking arrangements at St. Thomas's Hospital, ward-heating at the Samaritan Hospital for Women, and heating of an operating-theatre at the West London Hospital and of the out-patient department at the Cancer Hospital.

### Gifts and Bequests.

THE Right Hon. James Round, of Birch Hall, Essex, left £100 to the Essex County Hospital.

MISS JEMIMA HAYES, of Hereford, bequeathed £100 to the Hereford General Hospital and £50 to the Hereford Eye and Ear Hospital.

BEQUESTS under the will of Miss F. M. Cardwell include £1,500 to the Hostel of St. Luke, Fitzroy Square, and £300 to the Cheyne Hospital for Incurable Children.

MR. C. J. ATKINSON, of Bath, left £1,000 to the British Home for Incurables, Streatham, and £200 each to the Royal United Hospital and the Mineral Water Hospital, Bath.

MR. LOUIS DAVID BENJAMIN, of London, N.W., bequeathed £200 each to the Jews' Hospital and Orphan Home, Jews' Hospital for Incurables, and London Jewish Hospital, Stepney Green.

THE Chelsea Football and Athletic Club, Limited, have sent £6 10s. 10d. to the Royal Hospital for Incurables, Putney Heath, from the proceeds of the charity match Chelsea *v.* Fulham on May 5.

MR. W. H. SIDLEY, of Moseley, left the residue of his estate in equal shares to the Queen's and the General Hospitals, Birmingham, and specifically provided for the endowment of a bed at the last-named institution.

UNDER the will of Mrs. Zillah Wilcockson, of Eastwood, Nottingham, the Midland Institution for the Blind, Nottingham, receives £300, and the Nottingham General Hospital and Nottingham Children's Hospital £200 each.

MR. GEORGE GOODALL, of Lucknow Avenue, Nottingham, who died on March 15 last, left £100 each to the Nottingham General Hospital, Nottingham Children's Hospital, Nottingham General Dispensary, and Nurse Eva Kidd.

CUMBERLAND institutions benefit largely under the will of the late Mr. Robert Barton, of Carlisle. Amongst the bequests are £200 to the Convalescent Home, Silloth, £2,000 to the Cumberland Infirmary, £200 to the Carlisle Dispensary, and £200 to the Carlisle District Nursing Home.

MISS A. HOOPER, of Leamington, left £3,500 to the National Lifeboat Institution, to found and maintain a lifeboat. The residue of the estate is to be divided between the Birmingham and Midland Hospital for Women, the Warneford Hospital, Leamington, and the Railway Servants' Orphanage, Derby.

THE Rev. Prebendary H. W. Moss, for forty-two years head-master of Shrewsbury School, of estate to the gross value of £156,354 left one-tenth to charities. The bequests include £1,000 to the Salop Infirmary and £500 to the Eye, Ear, and Throat Hospital, Shrewsbury. Two thousand pounds was left to Shrewsbury School for a scholarship for boys needing assistance with their fees.

THE late Alderman Evan Evans, of Swansea, has left £1,000 to Swansea Hospital.

THE Walsall and District Hospital has been left £1,000 by the late Mr. George Gill.

MISS JANE WHITTINGHAM, of Surbiton, daughter of Mr. Charles Whittingham, of the Chiswick Press, left £500 to Guy's Hospital.

MR. STANSFIELD RICHARDSON, six times Mayor of Sunderland, left £500 to the Sunderland Infirmary, and other sums to local charities.

MR. HENRY TOWNSON, of Garth House, Wetheral, Cumberland, whose will has just been proved, left £500 to the Cumberland Infirmary, Carlisle.

MRS. SELINA ARTISS, of Hastings, left the residue of her estate to the Central London Ophthalmic Hospital, "not to be used for building or ornaments, but for poor afflicted ones."

UNDER the will of the late Mr. Thomas Tasker, £1,000 is bequeathed to the Batley and District Hospital "for the endowment of a bed to be called the Thomas and Martha Tasker bed."

MRS. CHRISTIANA BREWER left £300 to Brompton Hospital for Consumption, £200 each to Kilburn Dispensary and the British Home for Incurables, Streatham, and £100 to St. Andrew's Hospital, Clewer.

MISS SARAH WADDELL, of Bridgwater, has bequeathed £500 each to the Bridgwater Nursing Association, the Bridgwater Hospital, and the Devonshire Hospital, Buxton, and £250 to the Queen's Hospital, Birmingham.

OF the residue of the estate of Mr. Richard Blackburn, of Nottingham, the following hospitals at Nottingham will receive shares: The General Hospital, Children's Hospital, Samaritan Hospital for Women, the Hospital for Women, and the Institution for the Blind.

AFTER the death of his wife, one eighth of the estate of Mr. William Titterton, of Orpington, Kent, will pass under his will to the Royal Hospital for Incurables, one-eighth to the British Hospital and Home for Incurables, and two-eighths to Alderman Treloar's Home for Crippled Children.

MISS EMILY MORTIMER, of Niton, Isle of Wight, bequeathed £1,000 each to the Royal Isle of Wight Infirmary at Ryde and the Royal National Hospital for Consumption and Diseases of the Chest at Ventnor. The former institution will receive also a share in the residue of the estate.

MR. WILLIAM PRICE, M.B., of Sandymount, Southerndown, Bridgend, who died on January 11, left estate valued at £29,996 gross, with net personalty £27,153. After providing for annuities and other legacies, the testator left the residue of his estate to the University College of South Wales and Monmouthshire, for the Medical Department.

### THE NATIONAL RELIEF FUND.

The report of the Committee of the National Relief Fund to the President, H.R.H. The Prince of Wales, for the six months ending March 31, 1917, records that since the opening of the Fund £6,102,191 have been received for distribution, and that the balance in hand at the end of the half-year was £2,621,362. Although, happily, there has been an absence of any considerable distress amongst the civil population, the Fund continues to receive substantial public support, and contributions for the last twelve months for which the accounts have been published amounting to £165,726 were received; this source of revenue may diminish somewhat, but there is a considerable income from interest on sums on deposit or temporarily invested. As several branches of expenditure, such as the relief of distress amongst dependants of sailors and soldiers and help towards the provision of hospitals, are now within the sphere of work of other authorities, the present resources of the Fund, dealing as it does chiefly with civil relief, should be amply sufficient until any large change in economic conditions, should such change come about, is experienced.

### Where Civil Relief is Most Needed.

The East Coast towns, whose prosperity depends mainly upon fishing and the letting of lodgings, have suffered severely, and during the winter months the conditions in these towns were inevitably worse than in the preceding half-year. Amongst some of the local committees receiving grants for distribution from the Prince of Wales's Fund the following names and amounts may be mentioned: Great Yarmouth, £7,400; Lowestoft, £1,500; Margate, £5,150; and Ramsgate, £1,210. To the Borough of West Ham a sum of £3,500 was granted, this, no doubt, largely to provide relief for the victims of the explosion. Victims of air-raids have been helped also, in respect of the replacement of destroyed furniture. Cases of distress amongst the professional classes are dealt with by a special Sub-Committee. Here, again, the work of the Fund has been made easier by the constitution of another authority; for, although conditions have not materially altered, the National Service Department should be able to provide suitable employment for many professional men peculiarly affected by war conditions. Arrangements for dealing with cases of distress amongst professional women have been made through the Central Bureau for the Employment of Women, which body received £1,216 in the six months under review. In Scotland there has been practically no distress coming within the functions of the Fund, and in Ireland, where grants are administered by the Irish Local Government Board, a sum of £5,000 was provided for the relief of distress in Dublin.

### HOSPITAL AND INSTITUTIONAL RESULTS.

**Middlesex Hospital.**—The total income (including £11,419 legacies) amounted to £45,644, and the total expenditure to £49,864. The average weekly cost per occupied bed was £2 3s. 4d., being 2s. 2d. higher than in 1915. Speaking of the late secretary-superintendent, Mr. F. Clare Melhado, the report reads: "It may truly be said that the hospital as it stands to-day, its reputation for efficiency, and the true spirit of charity which is there for those who seek it, will remain a lasting monument to his memory."

**Catherine Gladstone Free Convalescent Home, Mitcham.**—This home was opened by Mrs. Gladstone in the year 1866, at Clapton, during the cholera epidemic at the East of London, and, moving to Snaresbrook, was transferred in 1900 to Mitcham. The total number of patients admitted last year was 977, and of these 859 were discharged as quite well or very much improved; 437 cases came from the London Hospital. Income, £2,872; expenditure, £2,778.

**Meath Home, Godalming.**—The home is for epileptic women and girls, and the eighty-one beds at Godalming and twenty-six at Hayling were constantly occupied during 1916. The occupants are employed on needlework and in making baskets. Total income (including £3,268 payments for patients), £3,989; total expenditure, £3,840.

**Colonial Nursing Association.**—The object of the Association is to provide trained nurses for hospital and private work in the British Colonies and Dependencies and amongst other British communities abroad. The total number of nurses employed in 1915 was 301. The report contains some excellent photographs illustrating this widely distributed work, of the nature of which an interesting map also gives an excellent idea.

**St. John's Hospital, Lewisham.**—Four hundred and eighty-two in-patients were treated, and 165 of these were sick and wounded soldiers. The committee "confidently state that the civil population did not suffer by the reception of such soldiers." The average cost of each in-patient per week was £1 13s. 8d. Total income, £4,147; total expenditure, £4,511.

**Surbiton Cottage Hospital.**—The hospital is supported by the Metropolitan Hospital Sunday and Saturday Funds and the League of Mercy. The total income (including a donation of £400 incorrectly placed under legacies) was £1,409, and the expenditure £1,161. The cost of provisions rose from £314 in 1915 to £343 in 1916.

**Kingseat Mental Hospital.**—The asylum is under the administration of the Aberdeen City District Board of Control. The Medical Superintendent states that "the war has had little effect on our admission rate: in fact it has directly decreased the number of adolescent males admitted." The maintenance rate per patient for the financial year was £31 8s. 6d.

**Cumberland and Westmorland Asylum.**—There were 505 male and 450 female patients in the asylum at the end of 1916. To the regret and with the best wishes of the committee, Miss Robinson, the matron, resigned her position after forty-four years of asylum work. The death-rate was 11.7 per cent., the highest for many years, but of those who died a quarter of the total number were over seventy years of age.

**St. Andrew's Convalescent Hospital, Clewer.**—The average cost of each patient per week under the heading of "Maintenance" was 19s. 8d., three-fifths of which was spent on provisions. The total income, including a legacy of £521, was £3,908, and the total expenditure £3,522.

### Manager's Notices.

**All letters on business matters must be addressed to The Manager, "The Hospital," 28 and 29 Southampton Street, London, W.C. 2.**

**Subscriptions**, which may commence at any time, are payable in advance. Cheques and Post-Office Orders should be crossed London County and Westminster Bank, Covent Garden Branch, and made payable to the Manager of THE HOSPITAL.

RATES OF SUBSCRIPTION (payable in advance).

| | | |
|---|---|---|
| **UNITED KINGDOM.** | | |
| Three Months | (including Postage) | 2s. 0d |
| Six Months | do. | 4s. 0d |
| Twelve Months | do. | 6s. 0d. |
| **FOREIGN AND COLONIAL.** | | |
| Three Months | (including Postage) | 2s. 6d. |
| Six Months | do | 5s. 0d. |
| Twelve Months | do. | 8s. 6d. |

**It is especially requested that remittances be made by Cheque or Postal Order, and in halfpenny stamps only when the amount is under sixpence.**

**Now Ready, "The Hospital" Index to Vol. LXI., Oct. 1916 to April 1917. Price 2½d. per copy post free.**

